# Supplementary material for: Structure and design of Langya virus glycoprotein antigens
Source: bioRxiv. 2023 Aug 26:2023.08.20.554025. Preprint. [Version 2] doi: 10.1101/2023.08.20.554025 (PMC10462157; doi:10.1101/2023.08.20.554025)
Supplement: Supplement 1 [file NIHPP2023.08.20.554025v2-supplement-1.pdf]

bioRxiv preprint doi: https://doi.org/10.1101/2023.08.20.554025; this version posted August 26, 2023. The copyright holder for this preprint (which was not certified by peer review) is the author/funder, who has granted bioRxiv a license to display the preprint in perpetuity. It is made available under aCC-BY 4.0 International license.

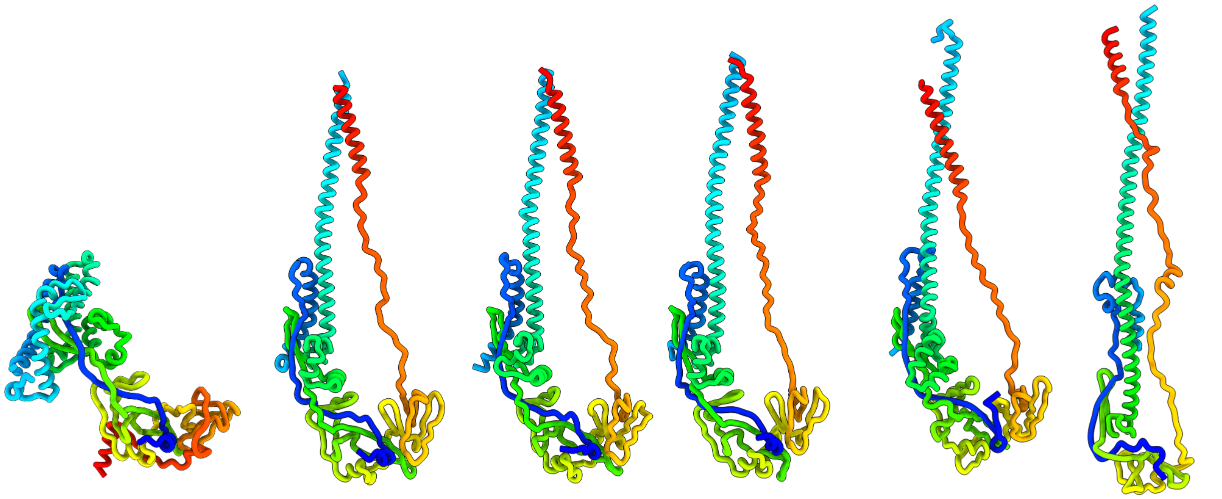

Figure S3 **Conservation of the general architecture of paramyxovirus and coronavirus fusion proteins.**

Ribbon diagrams of viral class I fusion proteins underscoring the architectural conservation among paramyxovirus and coronavirus postfusion structures. All models are colored using a rainbow scheme from blue (N-terminus) to red (C-terminus). The structures rendered are hPIV3 F: PDB 1ZTM; NDV F: PDB 3MAW; RSV F (PDB 3RRR); MHV S (PDB 6B3O).

bioRxiv preprint doi: https://doi.org/10.1101/2023.08.20.554025; this version posted August 26, 2023. The copyright holder for this preprint (which was not certified by peer review) is the author/funder, who has granted bioRxiv a license to display the preprint in perpetuity. It is made available under aCC-BY 4.0 International license.

Extended Data video 1. 3DFlex cryoEM density series oscillating between 41 frames showing movement of the LayV G stalk.
